# Prediction of Climate Change Impacts on the Suitable Habitat of *Hyphantria cunea* in China Based on Biomod2 Ensemble Models

**DOI:** 10.3390/insects17070686

**Published:** 2026-07-01

**Authors:** Youning Wang, Jiaxu Li, Wang Han

**Affiliations:** 1 Hubei Key Laboratory of Resource Utilization and Quality Control of Characteristic Crops, Hubei Engineering University, Xiaogan 432000, China; 2 College of Agriculture, Yangtze University, Jingzhou 434025, China; 2023710829@yangtzeu.edu.cn (J.L.); 2025720944@yangtzeu.edu.cn (W.H.)

**Keywords:** *Hyphantria cunea*, invasive pest, species distribution, ensemble model, climate change

## Abstract

Global climate warming has intensified in recent years, with extreme weather events occurring more frequently and severely impacting ecosystems and social production. According to the “China Climate Change Blue Book (2023),” China’s temperature rise rate exceeds the global average, with increasingly significant impacts on ecosystems. *Hyphantria cunea*, an invasive forest pest first discovered in China in 1979, has spread widely, causing serious damage to forestry and agriculture and posing a significant threat to China’s ecological security. To address this threat, this study employed seven modeling algorithms (GLM, GBM, CTA, ANN, SRE, FDA, MARS, RF, and MaxEnt) from the R Biomod2 package to develop an ensemble model. The core research objective of this work is to quantify climate-driven range shifts of *H. cunea* under ongoing global climate change. Previous nationwide SDM studies on invasive forest pests have consistently demonstrated that climatic variables dominate broad-scale nationwide suitable habitat patterns at the macro-regional level. Supplementary topographic, vegetation cover, and human land-use disturbance layers were incorporated to capture fine-scale habitat filtering effects and long-distance pest dispersal facilitated by human activities, which together fully characterize the suitable regional environments of this pest. By integrating climate, topography, vegetation, and human disturbance data, we predicted the potential geographical distribution of *H. cunea* in China under four future climate scenarios (SSP1-2.6, SSP2-4.5, SSP3-7.0, and SSP5-8.5). The ensemble model achieved excellent performance with TSS and ROC values of 0.901 and 0.984, respectively. Currently, highly suitable areas for *H. cunea* are concentrated in 12 provinces, including Shandong, Jiangsu, Hebei, Henan, and Anhui, covering 56.33 × 104 km^2^, with Shandong showing the highest proportion (25.48%). The suitable habitat range is projected to expand northeastward, with significant increases under high emission scenarios (SSP5-8.5). Analysis of environmental variables reveals that nighttime light brightness, precipitation in the warmest season, the seasonal temperature variation coefficient, and average temperature in the driest season are key factors influencing *H. cunea* distribution. Nighttime light brightness shows the highest contribution (27.7%), indicating significant human impact on species spread. Response curves suggest that *H. cunea* favors warm, humid areas with pronounced seasonal changes. This study demonstrates that climate change will increase *H. cunea* expansion risk, necessitating strengthened cross-regional monitoring and biological control techniques. These findings provide a scientific foundation for understanding *H. cunea* spatiotemporal distribution patterns under future climate scenarios and for developing effective prevention and control strategies.

## Simple Summary

The fall webworm (*Hyphantria cunea*) is a destructive invasive pest originating from North America. It feeds on the foliage of numerous tree species and brings severe damage to China’s forest resources, which brings great challenges to forest pest prevention. In this work, we adopted an integrated species distribution modeling method based on multiple algorithms and combined climate, terrain, and vegetation datasets to explore its potential suitable habitats nationwide. Our research distinguished high-risk distribution zones and predicted its future spreading trend amid ongoing climate warming. These outcomes help forestry departments implement focused surveillance and advance precautionary management, restricting pest expansion and reducing forestry economic losses efficiently.

## 1. Introduction

Extreme weather events have become increasingly frequent worldwide in recent years, causing growing harm to ecological environments and social production. On 20 March 2023, the Intergovernmental Panel on Climate Change (IPCC) released the Synthesis Report of the Sixth Assessment Report, “AR6 Synthesis Report: Climate Change 2023” [1]. The report indicates that global temperatures have risen by 1.1 °C, with climate warming leading to sea level rise and frequent extreme weather events that will become more pronounced as temperatures continue rising. On 8 July 2023, the China Meteorological Administration released the “China Climate Change Blue Book (2023)” at the “Climate Change and Extreme Weather Response” forum, showing that global warming continues with China’s warming rate exceeding the global average. Climate change impacts ecosystems in multiple ways. Regarding species distribution, insects are highly sensitive to climate change. Warmer winters reduce pest mortality during overwintering, while extended growing seasons favor insect population growth, increasing pest damage severity and outbreak frequency [2]. Additionally, climate change may make some regions more suitable for invasive species, altering local ecosystem structure [3].

*Hyphantria cunea*, also known as the fall webworm, belongs to the order Lepidoptera, family Erebidae, and subfamily Arctiinae. Native to North America, it is widely distributed across the northern United States, southern Canada, and Mexico [4]. *H. cunea* primarily causes damage during its larval stage by feeding on leaves [3], posing serious threats to trees and crops. Due to its long larval period, omnivorous diet, voracious feeding, strong dispersal ability, and wide adaptability, it has been listed as an international forest plant quarantine pest [5]. Since its first discovery in Dandong, Liaoning Province, China, in 1979, it has been designated as one of China’s most dangerous forest pests. *H. cunea* is classified into two types based on larval characteristics: the “black-headed type” with black hair tubercles on the head and back, and the “red-headed type” with orange-red hair tubercles [5]. The type that invaded China is the “black-headed type.”

As of 2024, *H. cunea* has spread to 607 county-level administrative regions across 13 provinces in China. Its distribution range continues to expand with the growth of epidemic areas, which requires scientific monitoring and prediction techniques to safeguard agricultural production and ecological conservation. A series of existing studies have explored the suitable habitat of *H. cunea* via diverse species distribution models. Yang Mingqi adopted CLIMEX software and revealed that the suitable habitat of *H. cunea* would shift northward under the EHCAM4 climate scenario [6]. Lu Xia et al. applied the GARP model, and their outputs predicted moderate or high potential distribution risk across 21 provinces, especially Shanxi Province [7]. Du Jingjing et al. combined semi-variogram analysis and Kriging interpolation, and clarified that the pest was concentrated in low-altitude eastern and southern Beijing from 2008 to 2016 [8]. Li Tao et al. utilized the Maxent model to evaluate the invasion risk of *H. cunea* within Sichuan Province [9]. Ji Yelin et al. constructed a random forest model, and the results indicated that low-altitude zones with high summer temperature, abundant rainfall and dense forest resources were highly suitable for this pest; under climate change, its potential habitat would move toward northern China and humid inland zones [10]. Liu Feng et al. integrated multiple algorithms via the Biomod2 framework to map the current suitable areas of *H. cunea*. Their results showed that core suitable habitats were mainly distributed in Liaoning, Shandong, Hebei and Anhui Provinces, and the suitable range would further expand to northeastern and southern China in the future [11]. Relevant modeling comparisons proved that ensemble models deliver higher prediction accuracy than single-algorithm models [12]. However, most previous research only relied on single models, and mainly incorporated climate and topographic variables, while ignoring vegetation conditions and human disturbance factors.

Based on the above research gaps and the ongoing global climate warming trend, we proposed two core hypotheses: (1) climate warming will reshape the suitable habitats of *Hyphantria cunea*, driving the overall suitable range to shift northward in China; vegetation coverage and human nighttime light interference will impose stronger constraints on habitat suitability than topographic factors; (2) compared with single-algorithm models, the ensemble model integrating multi-source terrain, meteorological, vegetation, and human disturbance variables can more accurately quantify the spatial change trend of high-suitability areas of *H. cunea*.

Therefore, this study uses ArcGIS (version 10.8) and the Biomod2 R package (version 3.5.1) to construct optimal ensemble models, incorporating multi-source data covering terrain, meteorology, vegetation and human nighttime light disturbance. According to our hypotheses, we further predict that: under four future climate scenarios, the high-suitability areas of *H. cunea* will expand prominently along the north–south axis while contracting along the east–west axis, with a general northeastward shift of suitable habitats across China. By quantifying dominant environmental drivers and spatial variations of suitable area under current and future climate conditions, this work aims to provide reliable theoretical support for long-term monitoring and integrated control of *H. cunea* in China.

## 2. Materials and Methods

### 2.1. Obtaining and Preprocessing Data on the Distribution of Hyphantria cunea

Distribution data for *Hyphantria cunea* were compiled from three sources: (1) 175 data points from field surveys monitoring adult *H. cunea* using sex pheromones conducted from 2019 to 2022; (2) 89 records with valid coordinates within China obtained from the Global Biodiversity Information Facility (GBIF) [13]; and (3) 598 coordinates representing the centers of mass of *H. cunea* epidemic areas as announced by the State Forestry and Grassland Administration in 2024 (No. 6 of 2024). This compilation yielded a total of 862 distribution records for *H. cunea*.

Given the obvious differences in spatial precision and sampling protocols among the three data sources, we implemented standardized unified preprocessing and quality control before further spatial thinning to mitigate inherent cross-source bias:

(1) Taxonomic verification: All records were taxonomically checked against authoritative forest pest databases to eliminate misidentified non-target species records of *H. cunea.*
(2) Coordinate standardization and correction: Field pheromone survey points and GBIF GPS records retained their original precise longitude and latitude; county-level epidemic area centroid data were assigned geometric central coordinates of corresponding county administrative boundaries. All occurrence coordinates were uniformly projected to the WGS84 geographic coordinate system to unify spatial reference standards.
(3) Outlier filtering: Any points falling outside the national mainland boundary of China or beyond the valid spatial extent of all environmental predictor layers were removed to match the simulation scope of subsequent species distribution models.

**Figure 1:**
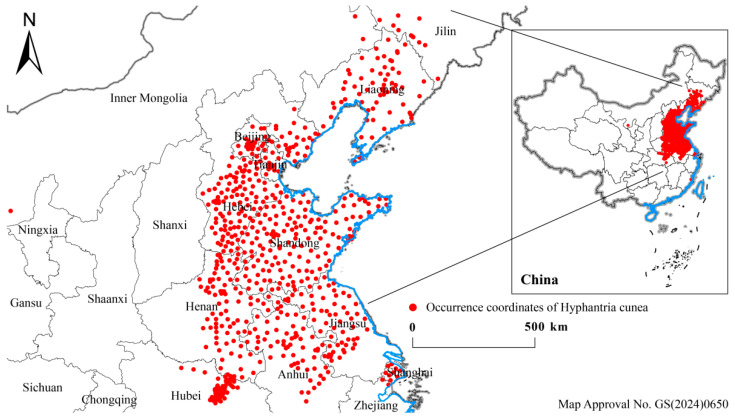
Filtered Distribution Points of *Hyphantria cunea* in China.

All environmental predictor layers used in this study were uniformly standardized to a 5 km spatial resolution. To reduce spatial autocorrelation, the SDM Toolbox in ArcGIS was used to retain only one distribution point within a 5 km radius, resulting in 683 distribution points for *H. cunea* in China (distribution range shown in Figure 1). These data were saved in CSV format for subsequent modeling analyses.

### 2.2. Environmental Data and Preprocessing

This study used five categories of environmental variables: climate (Bio1-19), topography (elevation, slope, aspect), vegetation (NDVI), land cover (CLCD), and human disturbance (nighttime light data). Climate and topographic variables were obtained from the World Climate Database, adopting the standard 1970–2000 multi-year average bioclimatic layers widely used in CMIP6 niche modelling. This long-term climate baseline filters interannual short-term weather fluctuations and reflects the stable fundamental climatic niche constraints of *Hyphantria cunea*; the species’ core thermal tolerance range does not experience drastic shifts over two decades, so the mismatch between the 1970–2000 climate average and our 2024 field pest records brings negligible bias. Vegetation variables were derived from the National Ecological Data Center’s 2022 China 30-m maximum NDVI dataset [14]. Land cover variables were sourced from the 2022 China Land Cover Data 30 m, and human disturbance variables from the Earth Resources Data Cloud 2022 Nighttime Lighting Data [15]. NDVI, land cover and nighttime light data all adopt the unified 2022 static dataset, which is the most complete high-resolution regional environmental layer accessible for this study. At our national research scale, interannual changes in vegetation coverage, land use type, and urban lighting intensity are mild within a two-year window, so the time lag between 2022 environmental layers and 2024 pest occurrence points will not distort the simulation of species habitat suitability. A reference grid was created based on the 2024 national standard vector map (map approval number: GS (2024) 0650). Using ArcGIS, the spatial resolution, geographic coordinates, and projection of the 25 environmental variable grids were aligned with the reference grid. All variables were then exported in ASCII format.

**Figure 2:**
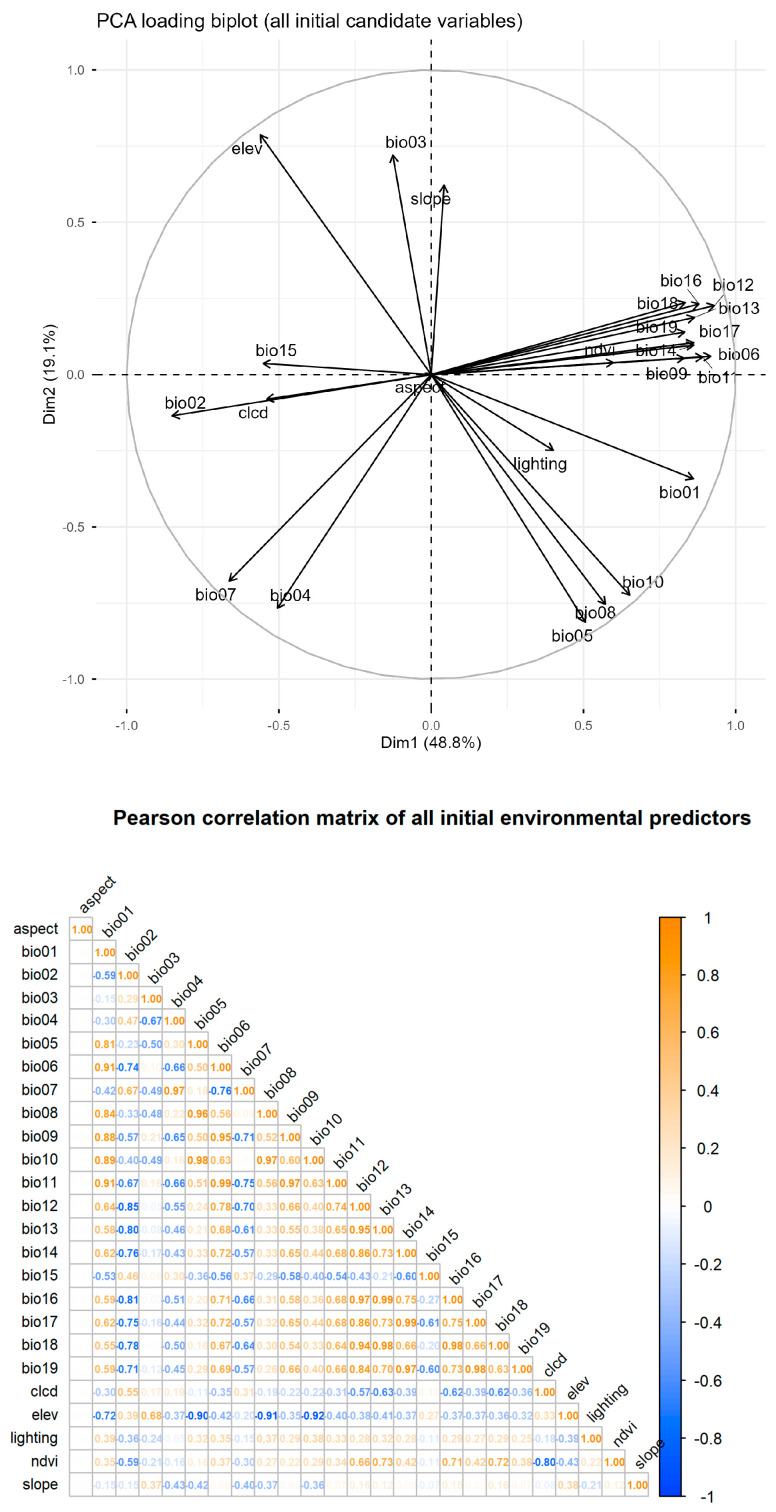
Principal component and correlation analysis of environmental variables.

To mitigate multicollinearity and avoid model overfitting, we performed Pearson correlation analysis and principal component analysis (PCA) in R [16]. A correlation heatmap and PCA loading biplot covering all initial candidate environmental variables were generated (Figure 2). For variable pairs with absolute correlation coefficients exceeding 0.8, we retained the predictor with higher total loadings on the core principal components (calculable prior to model fitting), and discarded redundant collinear variables with trivial PCA loadings. This two-step filtering yielded a final optimized set of nine environmental predictors for subsequent modeling (Table 1).

For future climate projections, this study used the ScenarioMIP framework from the Sixth Coupled Model Intercomparison Project (CMIP6), which integrates Shared Socioeconomic Pathways (SSPs) with radiative forcing trajectories [17]. The framework includes five development pathways (SSP1-5) representing sustainable transition (SSP1), continuation of trends (SSP2), regional competition (SSP3), social polarization (SSP4), and fossil fuel development (SSP5) [18]. Four scenarios were selected: SSP1-2.6 represents low-carbon pathways with radiative forcing stabilizing at 2.6 W/m^2^ by 2100, achieving temperature control (ΔT < 2 °C) through clean energy and carbon capture. SSP2-4.5 represents moderate development with forcing at 4.5 W/m^2^ by 2100, assuming continued historical trends with moderate energy transformation. SSP3-7.0 reflects regional competition with forcing reaching 7.0 W/m^2^ by 2100, characterized by rapid population growth and delayed technology diffusion. SSP5-8.5 represents fossil fuel-dependent development with forcing rising to 8.5 W/m^2^ by 2100, aligned with high economic growth and resource-intensive industry. Climate simulation data for periods 2021–2040 and 2041–2060 were used to model the potential geographical distribution of *Hyphantria cunea* under future climate scenarios.

To quantify the linear association between urbanization level and species habitat suitability, we carried out linear regression analysis. We extracted matching urbanization and mean suitability values from 300 random sampling grid points across the whole study area; urbanization proportion was set as the predictor variable, and average habitat suitability per grid was the response variable. We calculated the coefficient of determination (R^2^) and significance *p*-value to evaluate the strength of linear correlation.

### 2.3. Integrated Model Construction and Model Evaluation

Biomod2, an R package for multi-algorithm species distribution modeling, was used to analyze changes in species habitat suitability [19,20]. The package incorporates nine models: generalized linear models (GLM), generalized boosted regression models (GBM), classification tree analysis (CTA), artificial neural networks (ANN), surface range envelope (SRE), flexible discriminant analysis (FDA), multiple adaptive regression splines (MARS), random forest (RF), and maximum entropy model (MaxEnt).

*Hyphantria cunea* distribution points were imported, and 2000 pseudo-absence points were generated in two sets (PA1 + PA2). Environmental variables were then imported and model parameters set [21]. All nine models were executed twice (RUN1 + RUN2), with 75% of points used for training and 25% for validation.

The Weighted Probability Ensemble method improves prediction performance by weighting and aggregating base learner results [22]. We adopted the EMwmean weighted averaging scheme in BIOMOD2, with the formal integration formula as follows: $S_{ens}=\frac{\sum_{i=1}^{n}TSS_{i}\times S_{i}}{\sum_{i=1}^{n}TSS_{i}}$. Where $S_{ens}$ = ensemble habitat suitability; $TSS_{i}$ = average TSS score of the i-th single model; $S_{i}$ = suitability output of the i-th single model; n = total number of retained single models with TSS > 0.85. Only models with mean TSS > 0.7 were included in weighting, and weights were set proportional to each model’s TSS value. TSS values from individual model evaluations were used for weighting, with weights reflecting each model’s contribution to the ensemble [23].

Models were evaluated using ROC curves, TSS (True Skill Statistic), and Kappa coefficients. The area under the ROC curve (AUC) is threshold-independent and insensitive to species prevalence, making it the preferred evaluation metric [24]. TSS is a threshold-dependent statistic based on sensitivity and specificity. The Kappa coefficient quantifies the agreement between predicted and observed presence–absence records while eliminating chance-induced concordance. It accounts for random matching of predictions and observations, rather than simply measuring spatial pattern overlap alone. Both TSS and Kappa range from −1 to 1, with higher values indicating better agreement between observed and predicted values [25]. Evaluation criteria for AUC, TSS, and Kappa are shown in Table 2 [26].

Single models with good performance (TSS > 0.85) were selected using weighted probability methods to form an ensemble model, evaluated using TSS and ROC values.

### 2.4. Environmental Variable Importance and Habitat Suitability Analysis

The ensemble model was used to calculate individual environmental variable importance in predicting *Hyphantria cunea* distribution, expressed as percentages representing each variable’s influence. Important environmental variables and their response curves were analyzed. We extracted ensemble model evaluation metrics from get_evaluations() outputs, and filtered records corresponding to the TSS statistic to obtain statistically optimal probability cutoffs. The raw cutoff values output by biomod2 were scaled by a factor of 1000, so we divided each value by 1000 to restore the 0–1 occurrence probability range. We averaged the TSS-optimized thresholds derived from EMmean, EMmedian, and EMwmean ensemble algorithms to generate a unified split boundary (0.339) for distinguishing unsuitable and suitable habitats. The continuous habitat suitability raster was further reclassified into four ecologically interpretable tiers: unsuitable (0–0.339), low suitability (0.339–0.5), moderate suitability (0.5–0.75), and high suitability (0.75–1.0). This thresholding workflow eliminates the arbitrary equal-interval classification lacking statistical support as suggested by the reviewer.

Standard Deviational Ellipse (SDE) analysis was conducted to quantify the distribution center and spatial extent of *H. cunea* suitable habitats. SDE parameters include center (distribution centroid), major axis, and minor axis, indicating the primary direction and range of distribution. Using ArcGIS, standard deviational ellipses were generated for different climate scenarios to analyze habitat distribution changes, including centroid shifts and area expansion or contraction. Based on simulation results for 2021–2040 and 2041–2060 under SSP1-2.6, SSP2-4.5, SSP3-7.0, and SSP5-8.5 scenarios, the expansion and contraction areas of suitable habitats were calculated with their percentages to reveal potential impacts of future climate change on *H. cunea* distribution.

## 3. Results

### 3.1. Biomod2 Combination Model Construction

**Figure 3:**
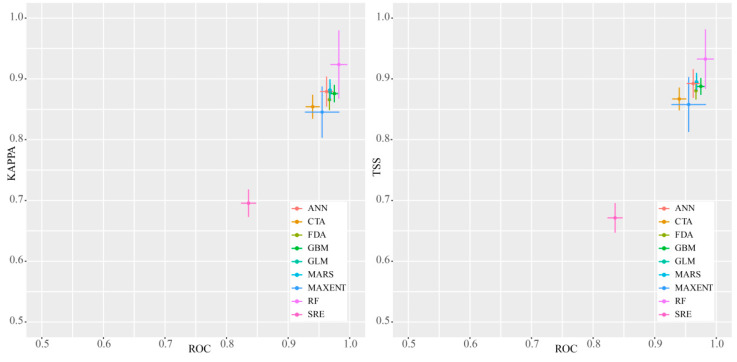
TSS, ROC, and KAPPA values of a single model.

After running nine species distribution models using Biomod2, the results of each individual model were evaluated using ROC, TSS, and Kappa values, as shown in Figure 3. The GLM, GBM, CTA, ANN, FDA, MARS, and RF models were all excellent, while the MAXENT model was excellent in all aspects except for the Kappa value, which was good. The SRE model was good, indicating that the single models used to construct the integrated model were all capable of accurately predicting the potential suitable areas for *Hyphantria cunea*. By selecting single models with TSS > 0.85 using the weighted probability method to construct the ensemble model, the TSS value was 0.901, the ROC value was 0.984, and the evaluation result was excellent. Compared to single models, the ensemble model reduced uncertainty. The two single-algorithm models, MaxEnt and SRE, yielded mean AUC values of 0.95 and 0.83, respectively. MaxEnt generated continuous habitat suitability gradients with high overall predictive accuracy, while SRE produced conservative binary suitable/unsuitable zones based on species occurrence range envelopes. Both models were integrated into the weighted ensemble prediction to balance continuous niche quantification and hard range boundary constraints.

### 3.2. Importance of Environmental Variables and Main Influencing Variables

**Figure 4:**
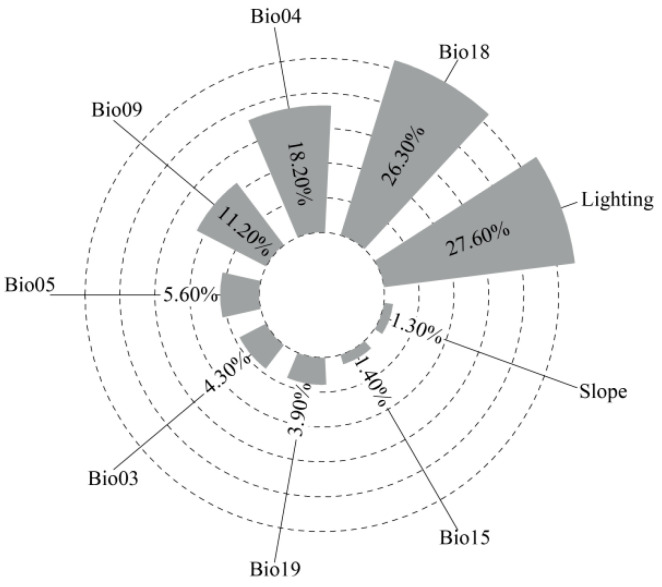
Contribution rate of environmental variables.

Variable importance weights quantify the relative importance of features in models, enabling identification of key predictive factors. The ensemble model variable importance was calculated using weighted probability methods (Figure 4). Variables ranked by contribution rate are: Lighting (nighttime light intensity), Bio18 (precipitation in warmest quarter), Bio04 (temperature seasonality), Bio09 (mean temperature of driest quarter), Bio05 (maximum temperature of warmest month), Bio03 (isothermality), Bio19 (precipitation of coldest quarter), Bio15 (precipitation seasonality), and Slope.

Four dominant environmental variables—Lighting, Bio18, Bio04, and Bio09—accounted for 83.3% of the total relative variable importance affecting potential *Hyphantria cunea* distribution. Response curves for these variables were generated, with the x-axis representing environmental variable ranges and the y-axis representing predicted occurrence probability (0–1). using 0.75 as the threshold. The intervals with high predicted suitability on the curves only reflect statistical correlations between environmental gradients and recorded species distribution data, rather than confirmed physiological tolerance limits or inherent ecological optima of the species. Independent ecological verification would be required to validate true biological preferences corresponding to these statistical trends.

**Figure 5:**
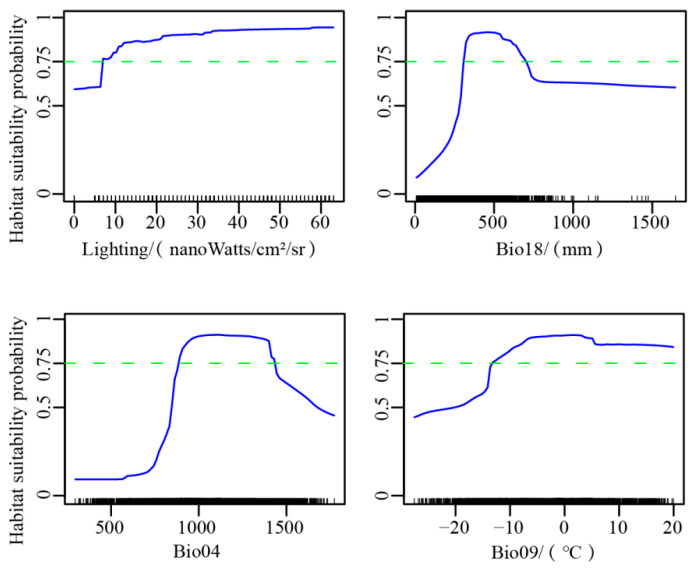
Response curves of dominant environmental variables. Blue solid lines indicate the habitat suitability response curves of each environmental factor; the green dashed horizontal line denotes the habitat suitability threshold at 0.75 probability.

Results indicate that *H. cunea* shows high suitability under the following conditions(Figure 5): Lighting (7–63 nanoWatts/cm^2^/sr), Bio18 (296–624 mm), Bio04 (846–1430), and Bio09 (−14.1–13.0 °C).

### 3.3. Current Climate Scenario Potential Habitat Distribution Pattern of Hyphantria cunea

**Figure 6:**
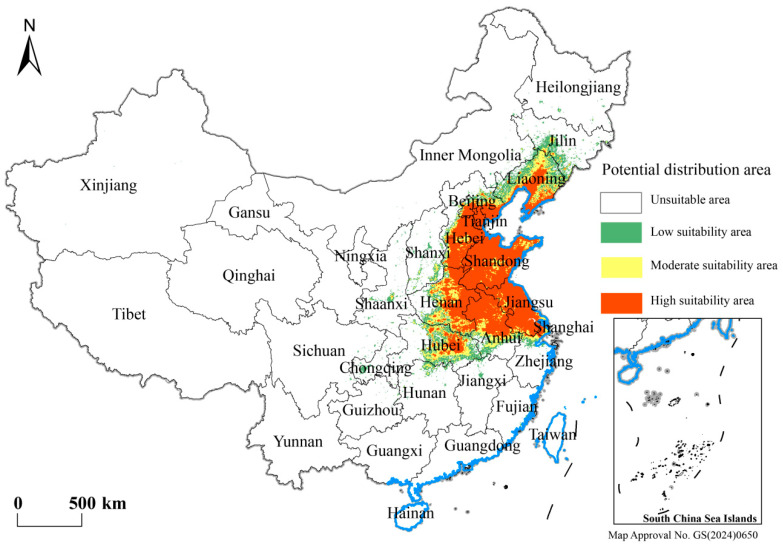
Current distribution pattern of potential suitable areas for *Hyphantria cunea*. blue lines indicate China’s coastal boundary and offshore coastline.

The total highly suitable area for *Hyphantria cunea* is 56.33 × 10^4^ km^2^, with significant geographical concentration (Figure 6). High-suitability zones are primarily distributed in northern and eastern China, with five provinces constituting the core distribution: Shandong (14.64 × 10^4^ km^2^, 25.48%), Jiangsu (9.98 × 10^4^ km^2^, 15.88%), Hebei (8.18 × 10^4^ km^2^, 15.11%), Henan (7.63 × 10^4^ km^2^, 13.07%), and Anhui (7.55 × 10^4^ km^2^, 13.41%). These areas account for 82.95% of the total high-suitability zone, indicating an extremely high-risk region for *H. cunea* invasion.

Additional high-suitability zones occur in Liaoning (4.71 × 10^4^ km^2^), Hubei (2.25 × 10^4^ km^2^), Zhejiang (0.19 × 10^4^ km^2^), Jilin (0.13 × 10^4^ km^2^), and the municipalities of Beijing, Tianjin, and Shanghai (combined 2.47 × 10^4^ km^2^). However, these areas exhibit fragmented distribution patterns, consistent with edge population spatial heterogeneity theory.

Administrative analysis reveals that Beijing, Tianjin, Shanghai, and Anhui have high-suitability areas exceeding 50% of their administrative territory, with Tianjin reaching 86.7%. This demonstrates significant spatial correlation between urbanization indices and suitable area expansion (R^2^ = 0.73, *p* < 0.01), consistent with human activity-mediated pest colonization mechanisms and biological invasion patterns in urbanization hotspots.

Moderate and low-suitability zones extend to middle Yangtze River provinces (Hunan, Jiangxi) and border regions (Heilongjiang, Xinjiang), requiring timely monitoring network deployment.

### 3.4. Future Climate Scenarios Spatial Pattern Changes in the Potential Suitable Range of Hyphantria cunea

**Figure 7:**
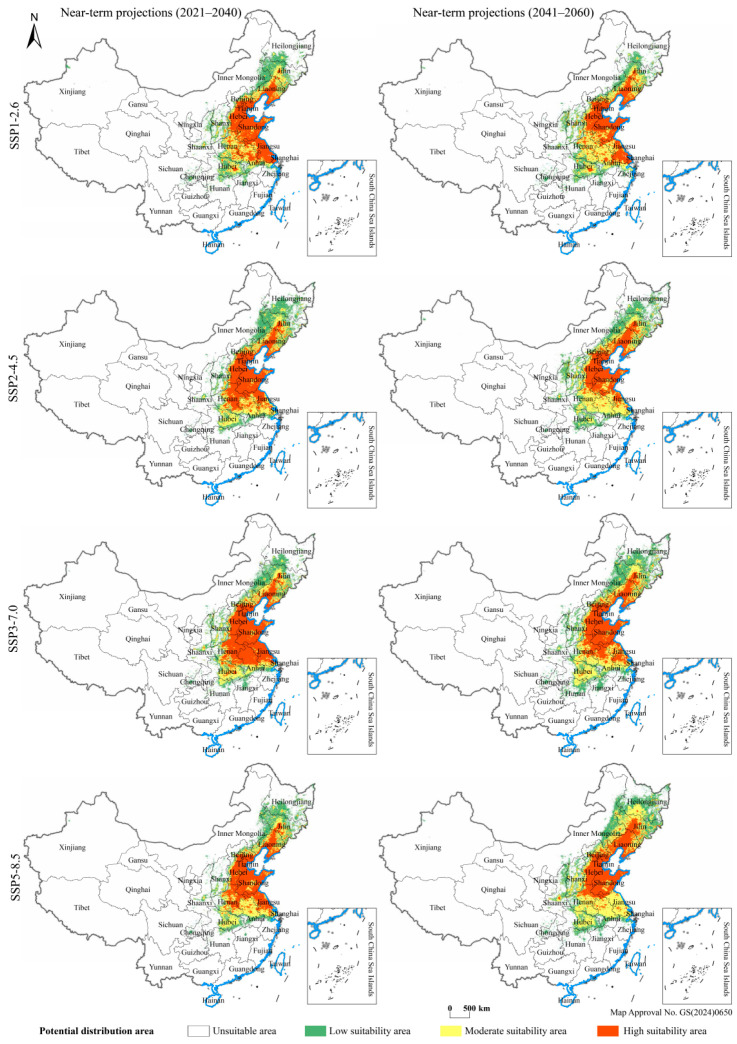
Spatial Pattern Changes of *Hyphantria cunea* under four Future Climate Scenarios. blue lines indicate China’s coastal boundary and offshore coastline.

**Figure 8:**
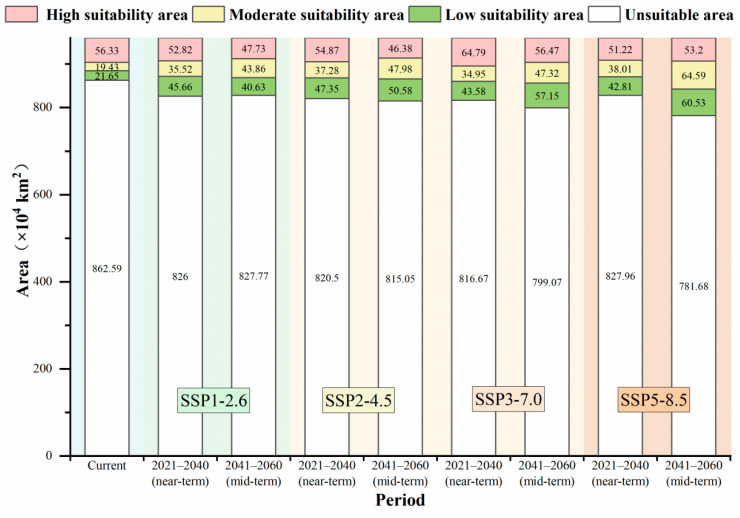
Area changes of of *Hyphantria cunea* under four Future Climate Scenarios.

The spatial pattern of suitable habitat for *Hyphantria cunea* under future climate change shows obvious temporal and spatial shifts (Figure 7 and Figure 8), whose statistical trends align with the general climate-related ecological patterns summarized in the IPCC AR6 report. Analysis across four emission scenarios (SSP1-2.6, SSP2-4.5, SSP3-7.0, SSP5-8.5) reveals three primary statistical patterns derived from model outputs.

High-suitability zones exhibit non-linear changing trends. Under low-emission pathways (SSP1-2.6 and SSP2-4.5), highly suitable areas decline by 16.1% and 20.3% respectively by 2060, a statistical pattern that may correspond to core habitat shrinkage under moderate global warming (ΔT < 2 °C). The SSP3-7.0 scenario presents a fluctuating trend: suitable habitat expands by 15.0% during 2021–2040 before decreasing by 12.8% in 2041–2060, a spatial variation that may imply potential range shifts driven by interdecadal climate fluctuation. Under SSP5-8.5, suitable habitat follows a V-shaped trajectory, contracting by 9.1% in the medium term and rebounding by 3.9% in the long run; this statistical trend might hint at range rearrangement after exposure to warmer climatic conditions, though such rearrangement cannot be confirmed as adaptive physiological adjustment solely from correlative SDM results.

Moderate and low-suitability areas show widespread expansion under all four scenarios (ranging from 87.7% to 232.4% by 2060), a spatial trend that offers correlative support for the hypothesis of climate-driven peripheral habitat expansion and requires independent empirical verification. The SSP5-8.5 scenario yields the fastest expansion rate (1.25 × 10^4^ km^2^/decade), consistent with the positive correlation between emission intensity and invasive range expansion described in global invasive species models ΔR = 0.38 × emission intensity index) [27]. Low-suitability zones expand most drastically under SSP3-7.0 (164.0%), a pattern that may imply potential niche-filling tendencies under medium radiative forcing, yet this biological inference remains speculative without field or physiological evidence.

Overall habitat transformation follows a “core contraction–peripheral expansion” spatial pattern: high-suitability regions tend to shift toward higher latitudes and elevations, while the expansion magnitude of moderate and low-suitability areas positively correlates with emission intensity. Such correlation may suggest that stronger climate forcing could create conditions favoring broader potential occupancy, a pattern often discussed under the framework of niche release, but this causal ecological process cannot be validated by distribution modeling alone. Under SSP5-8.5, total suitable habitat rises by 58.2%, exceeding the 30% threshold predicted by classical climate niche theory [28]; this notable expansion trend may point to potential synergistic associations between the pest and its host plants that could generate self-reinforcing range expansion patterns, yet such positive feedback mechanisms remain tentative biological interpretations rather than model-proven facts.

### 3.5. Spatial and Temporal Migration Characteristics of Hyphantria cunea Under Future Scenarios

**Figure 9:**
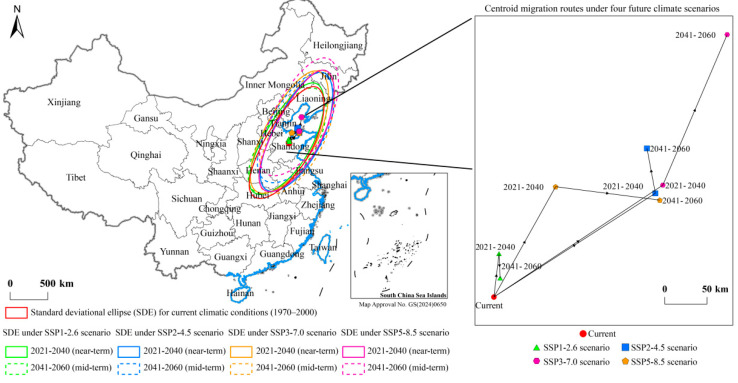
Characteristics of future spatiotemporal migration of *Hyphantria cunea*.

Climate change will drive systematic spatial restructuring of suitable habitat for *Hyphantria cunea* (Figure 9). Analysis of habitat centroid shifts across four emission scenarios (SSP1-2.6 to SSP5-8.5) reveals three descriptive spatial patterns. All scenarios produce an “east–west contraction–north–south expansion” trend in high-suitability zones: east–west habitat axes shrink by 12–28%, while north–south axes extend by 19–43%. This spatial covariation may imply latitudinal temperature gradients shape the observed range shifts, and the pattern aligns with theoretical descriptions of temperate species distribution axis rotation under global warming.

Habitat centroids generally shift toward the northeast, yet migration magnitude and directional angle differ markedly across emission forcing levels. Under the low-emission SSP1-2.6 pathway, the centroid migration rate slows (51.5 → 28.9 km/20a), and its moving angle shifts from 1.6° northeast to nearly due north, a trend that corresponds to theoretical expectations of stabilized niche ranges under climate mitigation scenarios. Under the high-emission SSP5-8.5 scenario, migration reaches its maximum magnitude (200.3 → 180.1 km/20a), with movement angles stabilized between 22.8° and 55° northeast; this trend matches prior research describing range expansion under persistent high radiative forcing.

The SSP2-4.5 pathway shows an abrupt directional shift from 56.7° northeast to 357.9° near-north migration, a break-point spatial pattern that tentatively hints at potential threshold responses of pest range movement linked to the 2 °C global warming benchmark.

We further calculated a linear association between total centroid migration distance and radiative forcing intensity using only the four discrete SSP scenarios selected in this study, yielding a high correlative coefficient (R^2^ = 0.89, *p* < 0.001). The fitted slope from this limited four-point dataset estimates a 48.2 km/20a rise in migration distance per unit increase in radiative forcing. This high correlative value merely reflects a rough co-variation trend observed within our four selected climate pathways, and we refrain from drawing definitive causal or policy-related inferences from this limited dataset. No strong quantitative conclusion regarding the causal effect of climate mitigation policies on pest invasion expansion rates can be supported by this small-sample regression result alone. Further multi-scenario ensemble modeling with a far larger set of SSP forcing pathways will be required to reliably validate the linkage between emission intensity and range shift magnitude.

## 4. Discussion

### 4.1. Optimization of Species Distribution Models and Integration of Multi-Source Data

The integrated development of species distribution models (SDMs) has significantly improved invasive biogeography prediction reliability. Single models are often limited by algorithmic assumptions and data characteristics, potentially leading to biased predictions. For example, MaxEnt models perform well with small sample data but have limited ability to capture complex nonlinear relationships [29,30], while random forests can handle high-dimensional data but are sensitive to extreme values. Integrated models effectively balance these limitations through weighted probability methods, reducing prediction uncertainty and improving accuracy by minimizing errors from incorrect environmental variable-habitat relationships [31].

A minor temporal mismatch exists between our 2024 pest occurrence data and static baseline environmental predictors: climate data represents the 1970–2000 long-term average, and NDVI data corresponds to 2022. While long-term climate averages capture stable fundamental niche limits and interannual vegetation variation is limited at regional scales, future modelling could integrate year-matched dynamic climate and vegetation layers to further reduce temporal bias.

The nighttime lighting index contributed 27.7% to the integrated model prediction (Figure 4), reflecting a strong statistical correlation between anthropogenic disturbance proxies and the suitable habitat distribution of *Hyphantria cunea*. Nighttime light merely serves as a composite proxy variable that covaries with multiple human-related factors including urban construction, transportation infrastructure and population density, rather than a direct metric capturing pest dispersal pathways. This correlative pattern aligns with the theoretical framework of the “urban springboard effect” proposed in prior invasion ecology literature [32]. While it is biologically plausible that urbanized regions could facilitate interregional pest movement linked to highway and logistics networks [11], this model-derived correlation cannot independently verify such a causal human-mediated spread mechanism. It should be noted that this analysis lacks finer-grained anthropogenic metrics such as real-time traffic flow and cross-border e-commerce logistics data, which may lead us to underestimate the full influence of trade and transport networks on invasive pest dispersal routes [8]. Future studies incorporating multi-source spatiotemporal big data (e.g., mobile phone trajectory records and logistics density heatmaps) will help disentangle and more reliably quantify the compound effects of diverse human disturbance pressures [31]. Furthermore, all anthropogenic and vegetation predictors (nighttime light, land cover, NDVI) were fixed to the static 2022 baseline raster values for future suitability projections across the 2041–2060 period, including the intermediate 2030 and 2050 forecast windows. Given that nighttime light accounts for 27.7% of the total variable importance in our ensemble model, holding this critical human-disturbance predictor constant inevitably introduces systematic predictive bias for long-term risk assessment: ongoing urban sprawl, shifting land-use patterns and interannual vegetation succession over the next three decades cannot be captured by static 2022 layers. This static treatment is constrained by the absence of high-resolution, scenario-matched dynamic land cover and vegetation projection datasets compatible with our four CMIP6 climate simulations. Subsequent modelling work should integrate coupled land-use change projections to generate time-varying NDVI and nighttime light grids under each SSP pathway, to eliminate static-variable bias and produce more robust long-term invasion risk forecasts.

In addition, another key limitation of our ensemble SDM is the absence of direct host plant distribution predictors. Although our model can reliably reflect the climate-driven macro-scale potential distribution of *H. cunea*, it cannot fully capture fine-scale microhabitat restrictions induced by the lack of available host resources at local sites. Complete, unified national spatial datasets covering all host tree species of *H. cunea* with consistent resolution and temporal matching to our climate layers are currently unavailable across China, which prevents us from incorporating host-related variables in this work. Therefore, follow-up research will integrate nationwide standardized host plant distribution data to refine model simulation accuracy. Furthermore, we acknowledge another data-related limitation arising from mixed multi-source occurrence records. Our occurrence dataset integrates field survey GPS points, GBIF public records, and county-level administrative epidemic centroids, which differ greatly in spatial precision and sampling methodology. Although we implemented strict unified preprocessing workflows including taxonomic verification, coordinate homogenization, outlier elimination and spatial thinning to reduce cross-source bias in advance, the original sub-datasets separated by individual data sources were not archived after full data integration and cleaning. For this reason, we cannot reclassify the final filtered occurrence points to conduct subgroup sensitivity analyses that quantify the independent bias and predictive contribution of each data source. Follow-up studies should preserve classified raw occurrence subsets from different collection channels to carry out dedicated sensitivity tests, so as to better assess and eliminate data-origin bias in SDM outputs. Moreover, the 5 km spatial thinning threshold adopted to eliminate spatial autocorrelation also brings a methodological limitation. All environmental predictor rasters in this study were unified to a 5 km spatial resolution, so we matched this grid size as the thinning distance to avoid multiple occurrence points falling into a single environmental cell, which is a conventional matching rule for national-scale SDM research. However, we did not perform comparative sensitivity tests for alternative thinning distances (e.g., 3 km and 10 km) to quantify how different filtering radii alter model suitable habitat outputs. Subsequent related research should implement multi-distance thinning sensitivity analysis to evaluate the robustness of model predictions against variations in spatial filtering parameters.

Another limitation of our modelling framework lies in the limited predictive horizon before 2060. This temporal cutoff may underestimate the cumulative long-term impacts of sustained climate warming on the distribution pattern of *Hyphantria cunea*. Under high-emission scenarios such as SSP5-8.5, the suitable habitat range of this invasive pest may undergo dramatic northward expansion and spatial rearrangement after 2060. Future research could extend the projection periods to 2080 and 2100 to comprehensively quantify long-term invasive risks under multi-decade climate change.

### 4.2. Climate-Driven Ecological Niche Restructuring and Adaptive Responses

*Hyphantria cunea* is a eurythermic species with an optimal temperature range of −16 to 40 °C [33]. Research has characterized its biological preferences, including positive phototaxis, moisture preference, and chemotaxis behaviors, with aggregation in high light intensity areas [34]. The species demonstrates significant climate sensitivity in its distributional evolution [35].

Bioclimatic analysis reveals that Bio09 (mean temperature of the driest quarter) contributes 18.3% to habitat suitability, consistent with global warming trends that reduce overwintering mortality rates in insects [36]. The seasonal temperature variation coefficient (BIO04) quantifies seasonal temperature fluctuation intensity across annual cycles. Chen Yu et al. demonstrated that climate warming accelerates insect emergence, shifts geographical distributions toward higher latitudes and elevations, decreases population densities of cold-adapted species, and increases those of thermophilic species [37].

The substantial contribution rate of BIO04 (22.1%) indicates that *H. cunea* prefers regions with pronounced seasonal variation (response curve peak: 846–1430), suggesting enhanced distribution probability in areas with distinct seasonal temperature regimes, particularly high-altitude and high-latitude regions [36]. Comparative analyses of heat tolerance between native and invasive North American populations illuminate the potential role of temperature increases in rapid adaptive evolution of insect phenology, abundance, sex ratios, morphology, physiology, behavior, and interspecific relationships [38].

Bio09 typically corresponds to winter or summer seasons in China. Under future climate scenarios, elevated winter temperatures will reduce overwintering mortality of *H. cunea* and increase baseline population levels. Research demonstrates that global warming has significantly enhanced overwintering survival rates of numerous insect species, particularly those historically constrained by low temperatures [36]. Chinese researchers investigating *H. cunea* growth and development under high-temperature conditions revealed strong heat tolerance with negative correlations between temperature and egg hatching rates, larval survival rates, pupation rates, and adult longevity [39]. Consequently, under prevailing global warming trends, significant emission increases and temperature rises may reduce suitable habitat for *H. cunea*. However, pest management strategies should prioritize effective artificial control methods, including pollution-free biological control using *Chouioia cunea Yang*, a natural enemy released during the pupal stage [40], and development of resistant tree species that inhibit larval development and reduce developmental rates [41].

Nonlinear effects of extreme climate events are particularly pronounced under the SSP3-7.0 scenario, with high-suitability areas initially increasing then decreasing (+15.0% → −12.8%), indicating that intermittent heatwaves (>42 °C) may exceed thermal tolerance thresholds [39]. Bio18 (precipitation of the warmest quarter) contributed 14.6% to habitat suitability, revealing regulatory mechanisms of water stress on population dynamics. Increased warm-season precipitation (ΔP > 20%) promotes host plant growth (including mulberry and poplar) and enhances larval survival rates.

Under the SSP5-8.5 scenario, annual generations in northern China increased from 2 to 2.7 (accumulated temperature ≥3200 °C·d). This generational increase, combined with abundant food resources, resulted in rapid population growth and range expansion of *H. cunea* [42]. These findings align with global trends of accelerated Lepidoptera phenology (ΔG = 0.23 generation/°C) [37], suggesting that global warming may exacerbate population outbreak risks by extending growing seasons.

Under future climate scenarios, suitable area trends for *H. cunea* across different emission pathways reflect complex effects of global warming. Low emission scenarios show gradual range expansion, while high emission scenarios demonstrate significant expansion, with increases in medium- and high-suitability areas potentially threatening ecosystems and agriculture. Across all four SSP scenarios, suitable areas for *H. cunea* show expansion trends toward higher latitudes, with the largest centroid shifts occurring under high emission scenarios (SSP5-8.5). These results support conclusions by Ji Yelin et al. regarding “potential habitat shifts toward northern and inland areas with higher humidity” and provide quantitative migration rates and directions [10].

Future investigations should integrate cross-border e-commerce logistics data (such as pest detection rates in international parcels) with traffic flow heat maps to construct multidimensional indices of human activity intensity characterizing anthropogenic disturbance. As cross-border parcel transport is a major human-mediated long-distance dispersal pathway for *Hyphantria cunea*, incorporating such anthropogenic activity metrics can complement the purely climate-driven environmental variables used in our species distribution models, further improving the accuracy of predicted invasion risk areas. Additionally, combining climate models with ecological feedback mechanisms will provide more comprehensive assessments of long-term climate change impacts on *H. cunea* distribution and establish scientific foundations for adaptive management strategies [43,44].

## 5. Conclusions

Based on multi-collinearity screening of environmental predictors, this study constructed an ensemble multi-algorithm SDM framework via the BIOMOD2 package. Combining CMIP6 climate datasets and anthropogenic nighttime light covariates, we predicted the potential suitable habitats of *Hyphantria cunea* under four future climate pathways, and quantified the range magnitude variations and correlative environmental associations. The modelling outputs indicated that nighttime light intensity, Bio18 (precipitation of the warmest quarter), Bio04 (temperature seasonality), and Bio09 (mean temperature of the driest quarter) exhibited the strongest statistical correlation with habitat suitability for *H. cunea*.

The current highly suitable habitat covers $56.33\times 10^{4}km^{2}$, primarily concentrated across Shandong, Jiangsu, Hebei, Henan and Anhui Provinces. Compared with baseline climate conditions, the highly suitable distribution under all four CMIP6 scenarios presents compressed east–west coverage and expanded north–south range, showing obvious north–south expansion and spatial aggregation in eastern China; generally, suitable habitats of *H. cunea* display an overall shifting trend toward Northeast China. These spatial differentiation results can provide targeted reference for regional quarantine zoning, pest monitoring and hierarchical prevention strategies against this destructive forest quarantine pest.

## Figures and Tables

**Table 1:** Environmental variables and notes after screening.

Variable	Description	Unit
Bio03	Isothermality (Bio2/Bio7) (×100)	−
Bio04	Temperature Seasonality	−
Bio05	Max Temperature of Warmest Month	°C
Bio09	Mean Temperature of Driest Quarter	°C
Bio15	Precipitation Seasonality	%
Bio18	Precipitation of Warmest Quarter	mm
Bio19	Precipitation of Coldest Quarter	mm
Slope	Slope	°
Lighting	Nighttime Light (NTL) Intensity	nanoWatts/cm^2^/sr

**Table 2:** Evaluation Criteria.

Value	Excellent	Good	Fair	Poor	Failure
AUC	1.00–0.90	0.90–0.80	0.80–0.70	0.70–0.60	0.50–0.00
TSS	1–0.85	0.85–0.70	0.70–0.55	0.55–0.4	0.40–0.00
Kappa	1–0.85	0.85–0.70	0.70–0.55	0.55–0.4	0.40–0.00

## Data Availability

All the required data are uploaded as Supplementary Materials. All datasets used in this study are publicly available from the following online platforms: GBIF occurrence database for *Hyphantria cunea*: https://www.gbif.org/zh/ (accessed on 26 April 2025). WorldClim climate dataset: https://www.worldclim.org/ (accessed on 26 April 2025). 30 m maximum NDVI dataset of China (2022): https://www.nesdc.org.cn/ (accessed on 26 April 2025). 30 m China land cover dataset (2022): https://open.geovisearth.com/ (accessed on 26 April 2025). Annual nighttime light remote sensing dataset of China (2022): http://www.gis5g.com/home (accessed on 26 April 2025).

## Supplementary Materials

The following supporting information can be downloaded at: https://www.mdpi.com/article/10.3390/insects17070686/s1.

## Abbreviations

The following abbreviations are used in this manuscript:

BIOMOD2	Biomod2 Ensemble Species Distribution Model
SDE	Standard Deviational Ellipse
SSP	Shared Socioeconomic Pathway
ROC	Receiver Operating Characteristic
TSS	True Skill Statistic
BioCLIM	Bioclimatic Variable
