# Supplementary figures and images for: Prediction of Climate Change Impacts on the Suitable Habitat of Hyphantria cunea in China Based on Biomod2 Ensemble Models
Source: Insects. 2026 Jul 1;17(7):686. doi: 10.3390/insects17070686 (PMC13410028; doi:10.3390/insects17070686)

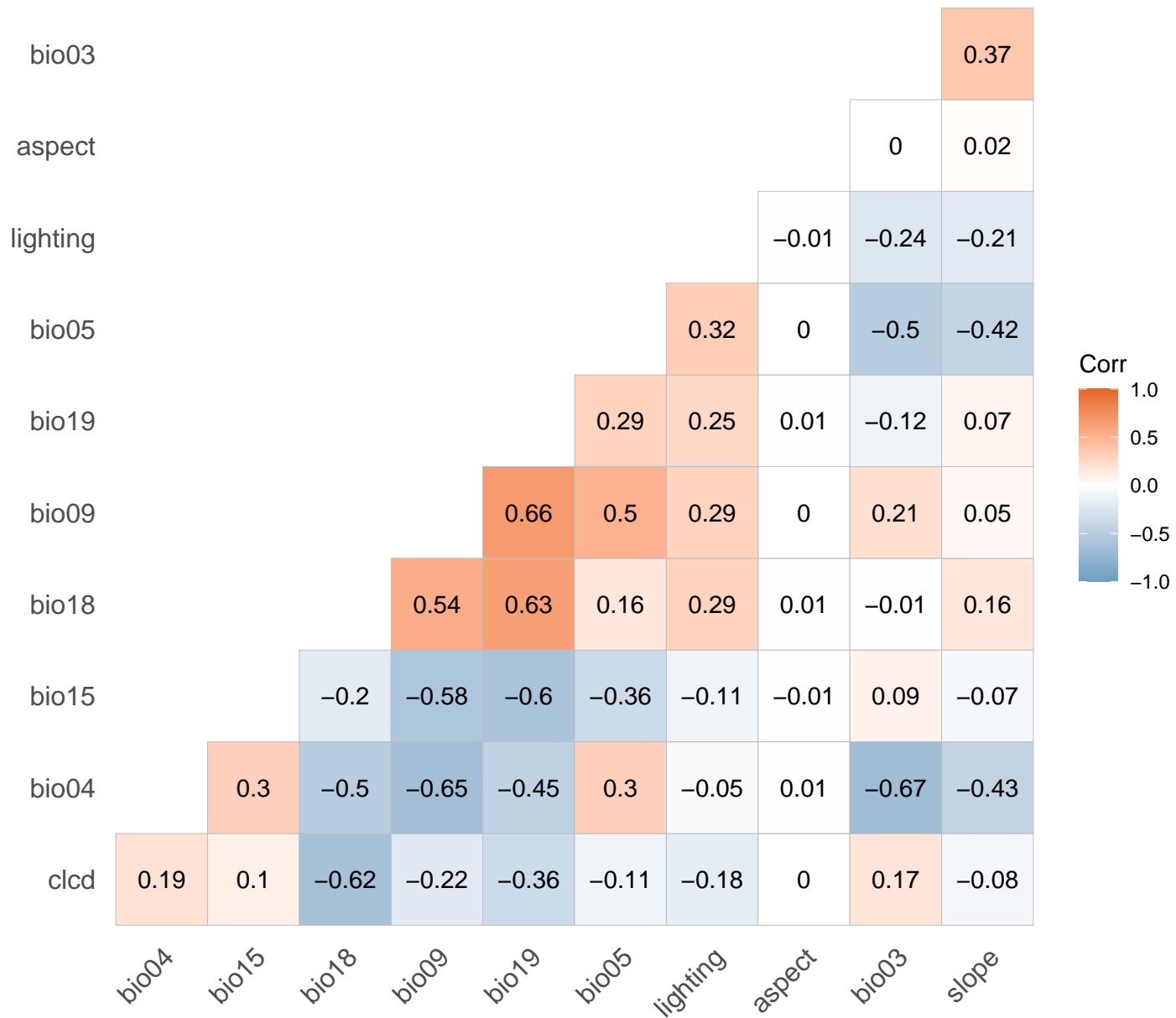

Supplement: Supplementary file 1 [file insects-17-00686-s001.zip › 04Picture/02correlation analysis.pdf]

Variables – PCA

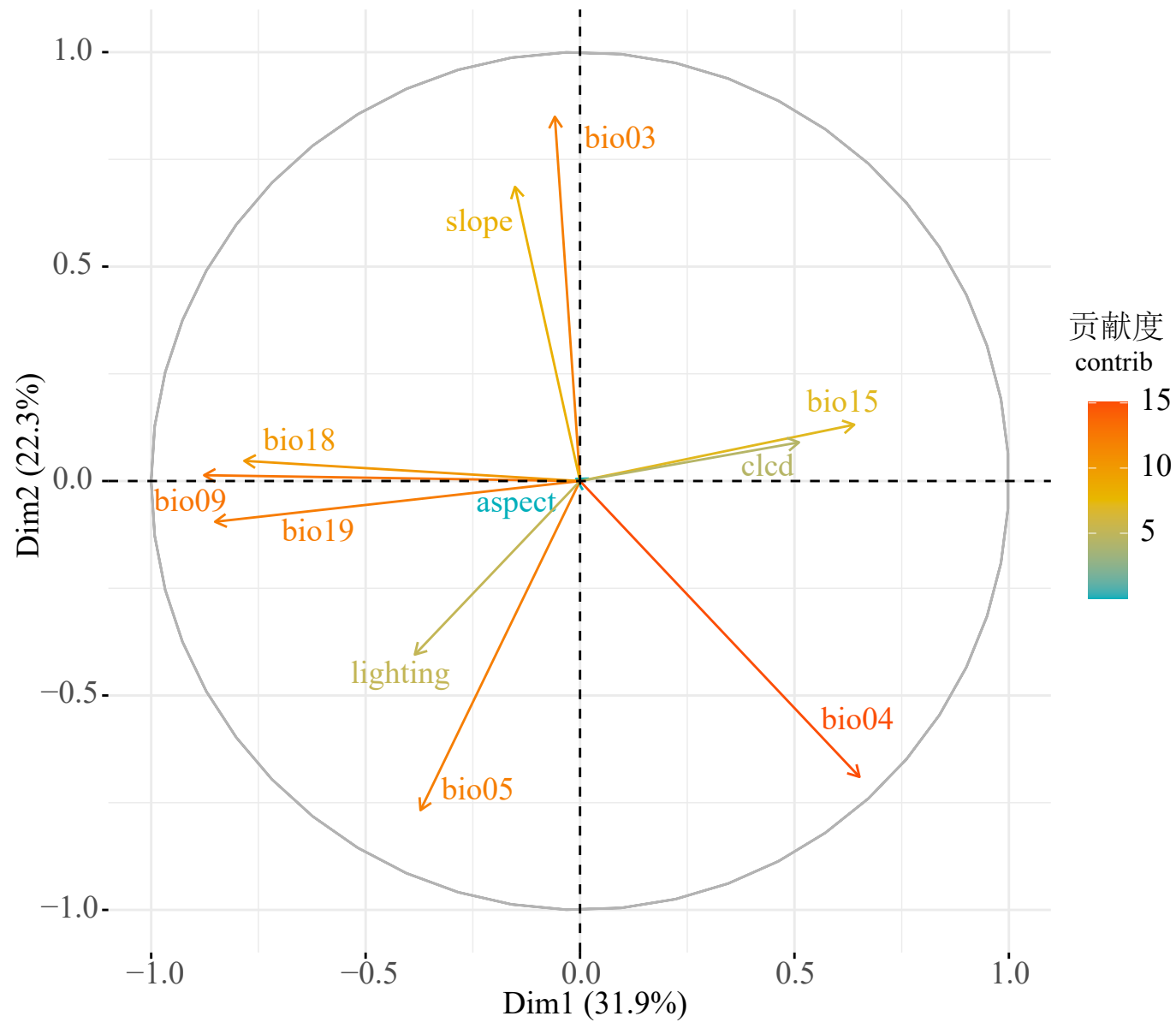

Supplement: Supplementary file 1 [file insects-17-00686-s001.zip › 04Picture/02pca.pdf]

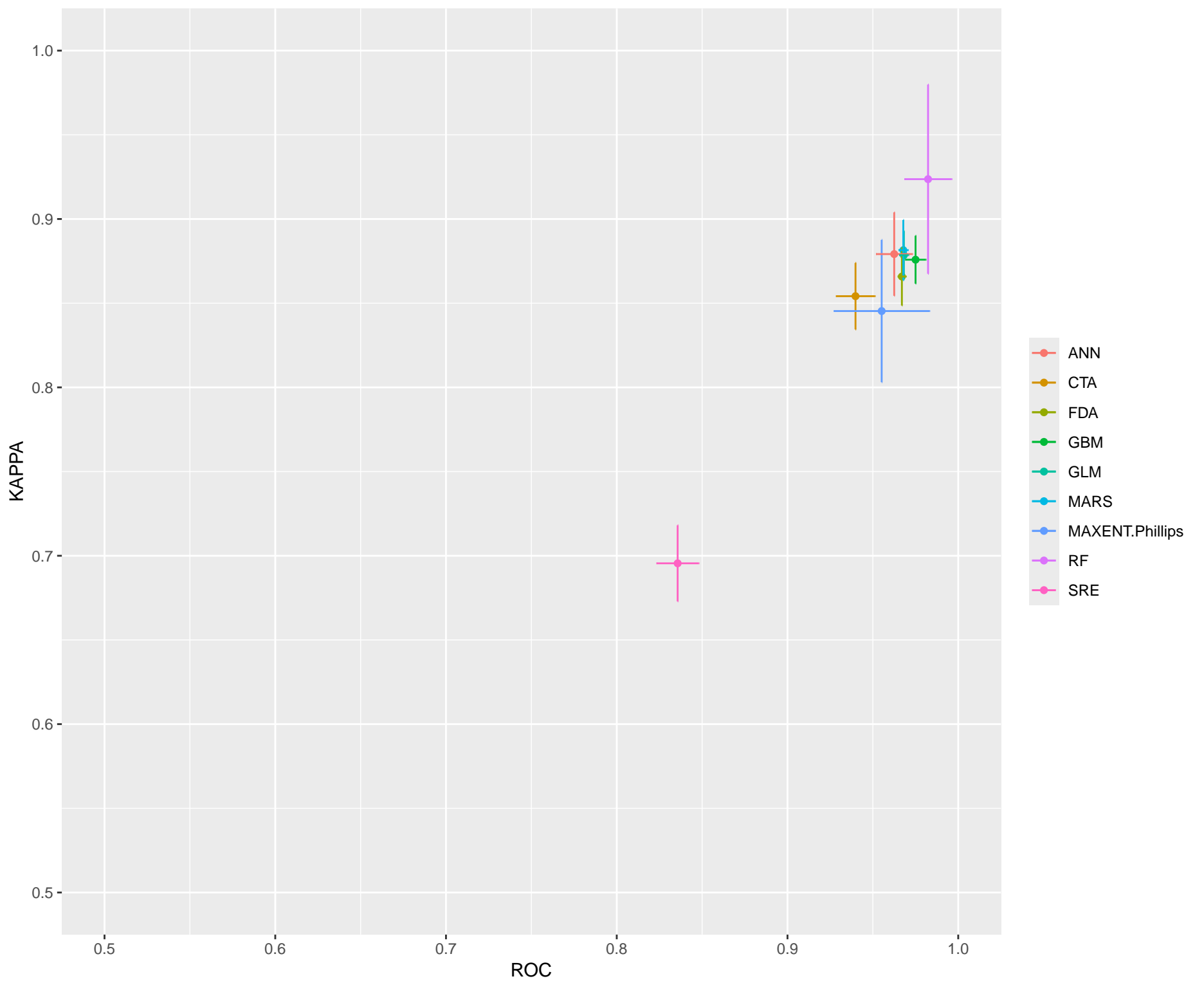

Supplement: Supplementary file 1 [file insects-17-00686-s001.zip › 04Picture/03kappa.pdf]

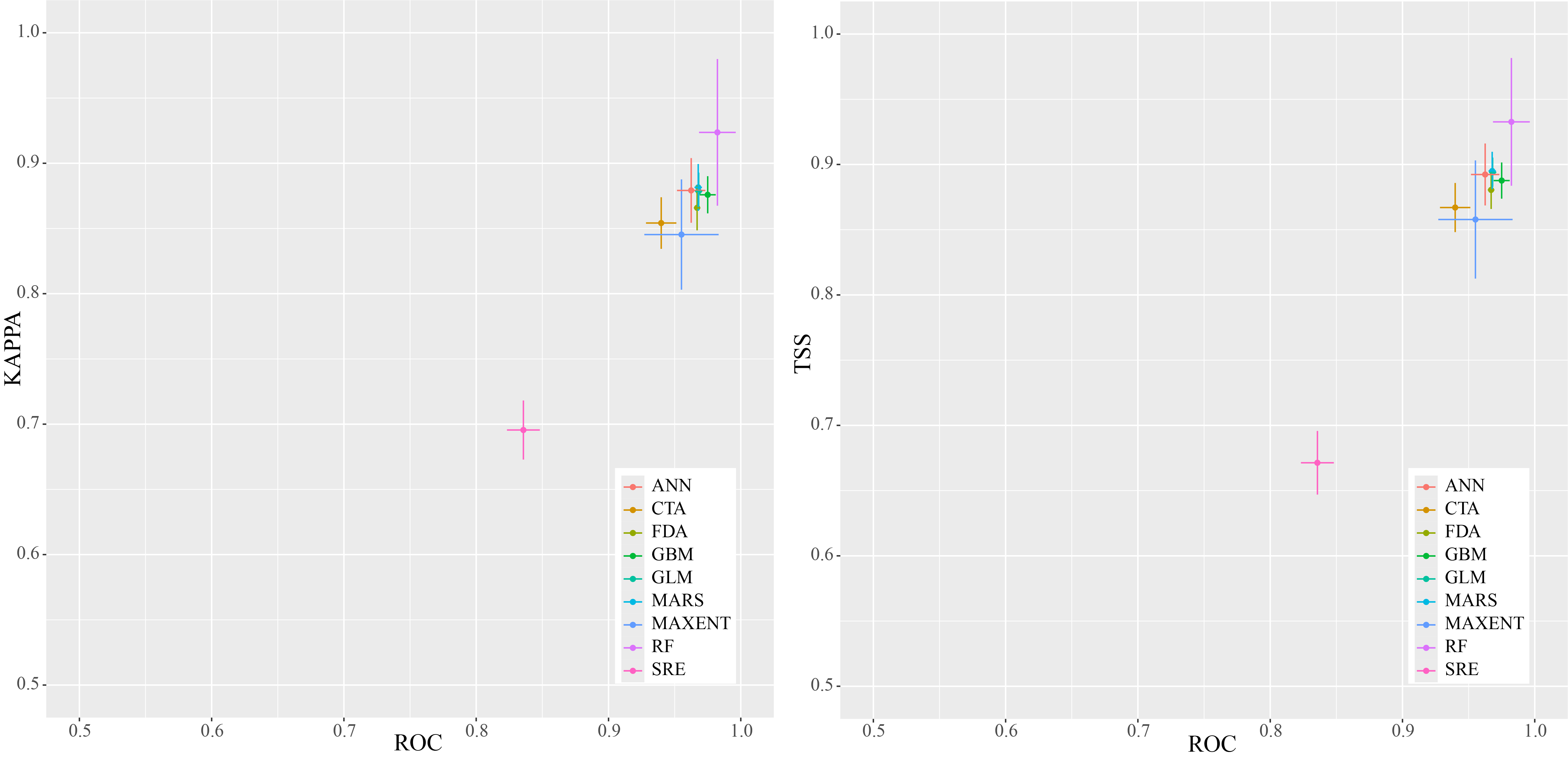

Supplement: Supplementary file 1 [file insects-17-00686-s001.zip › 04Picture/03TSS, ROC and KAPPA values of a single model.jpg]

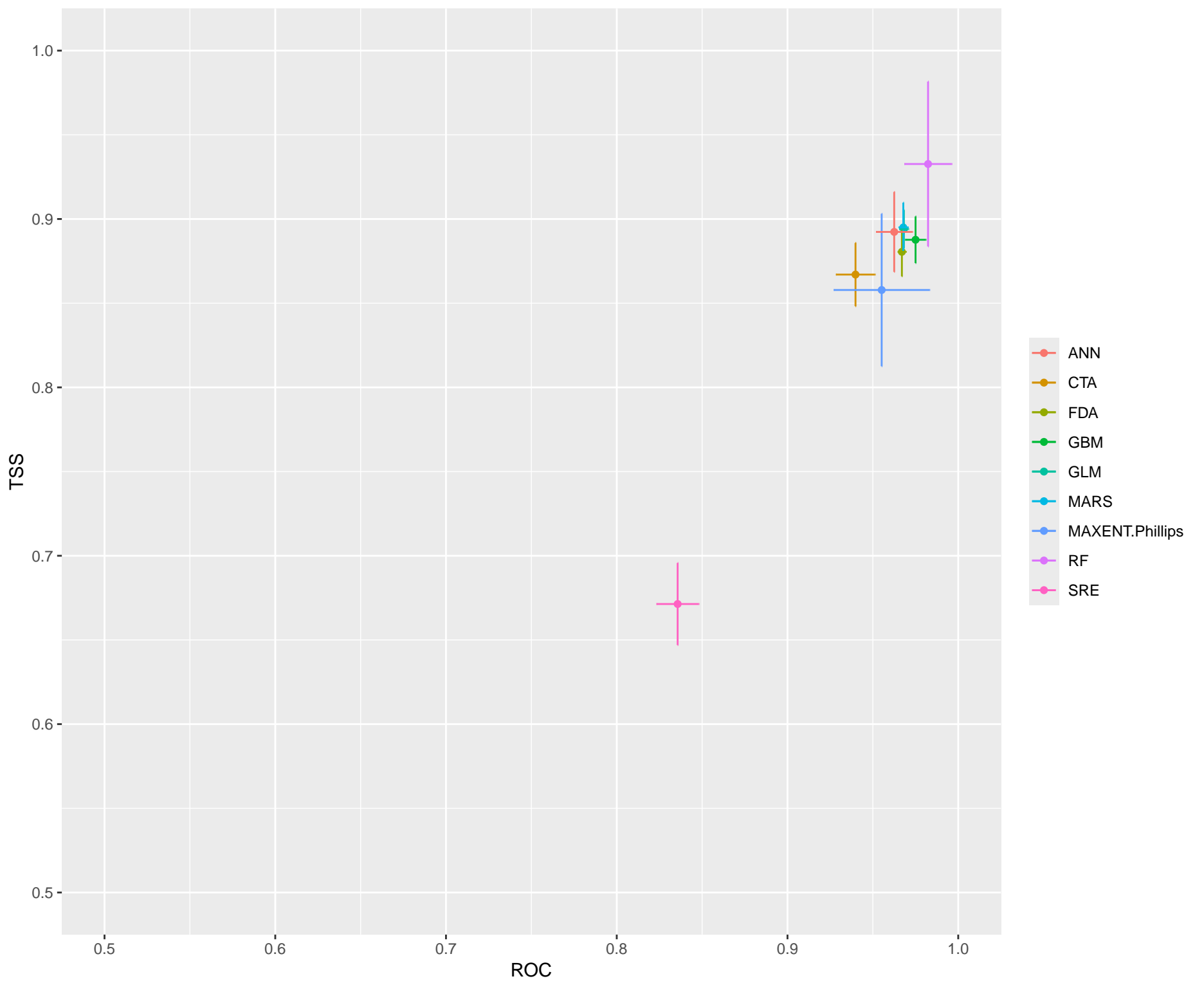

Supplement: Supplementary file 1 [file insects-17-00686-s001.zip › 04Picture/03tss.pdf]

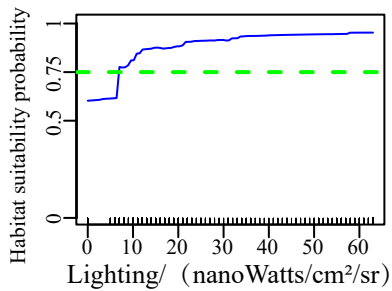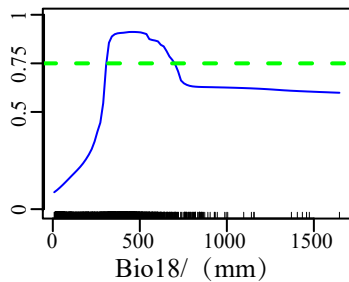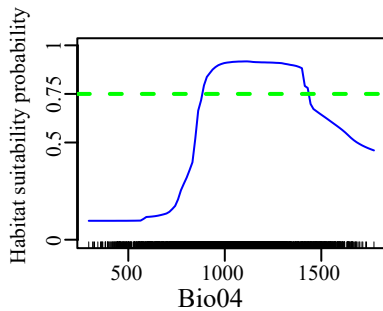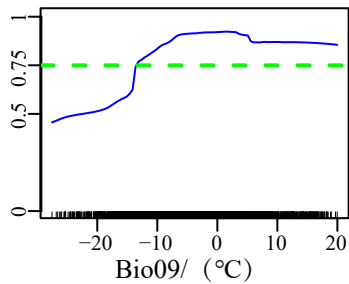

Supplement: Supplementary file 1 [file insects-17-00686-s001.zip › 04Picture/05Response curves of dominant environmental variables.pdf]

# Response curves for Presences.Absences's EMwmean

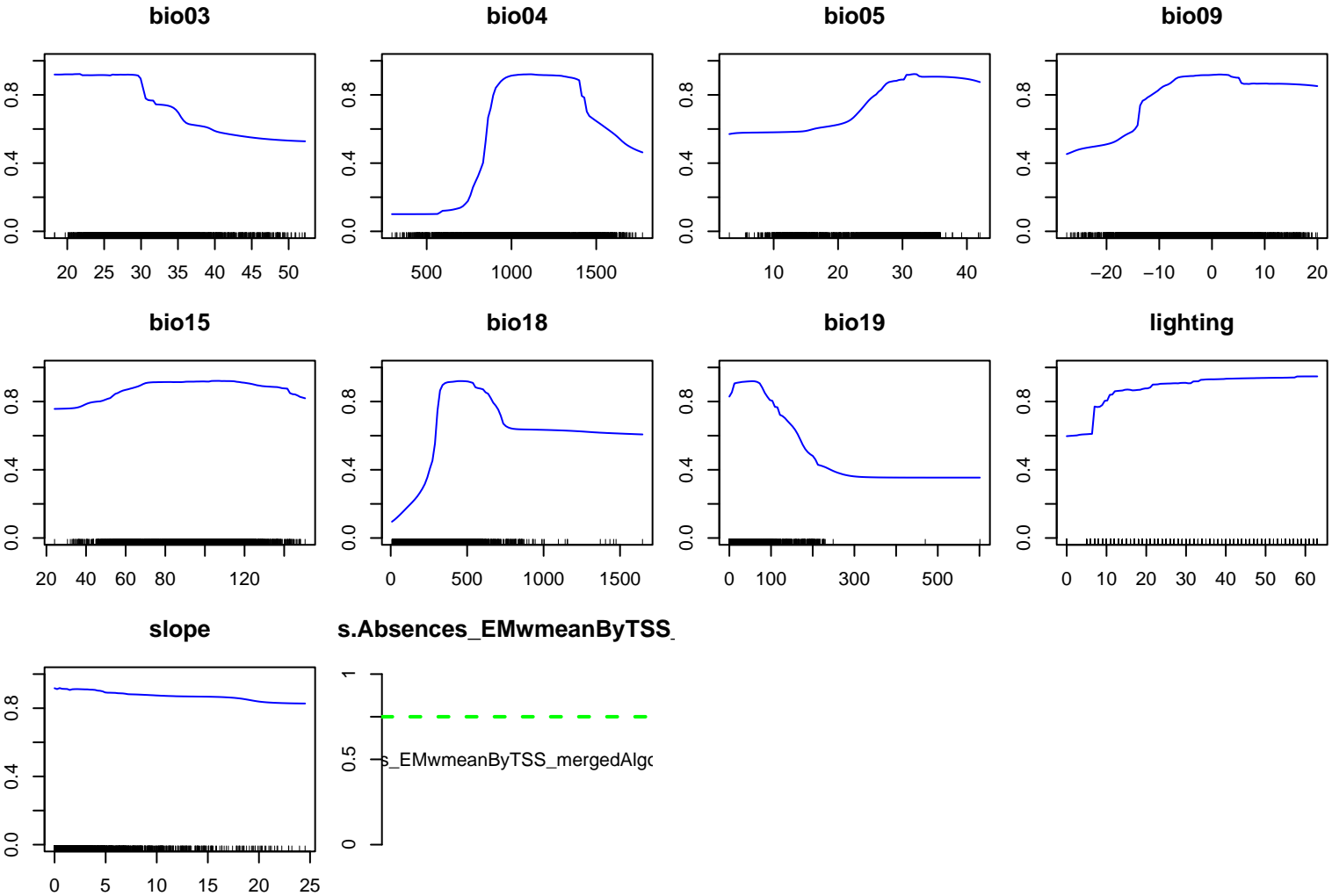

Supplement: Supplementary file 1 [file insects-17-00686-s001.zip › 04Picture/05Response_Curve_Presences.Absences_EMwmeanByTSS_mergedAlgo_mergedRun_mergedData.pdf]

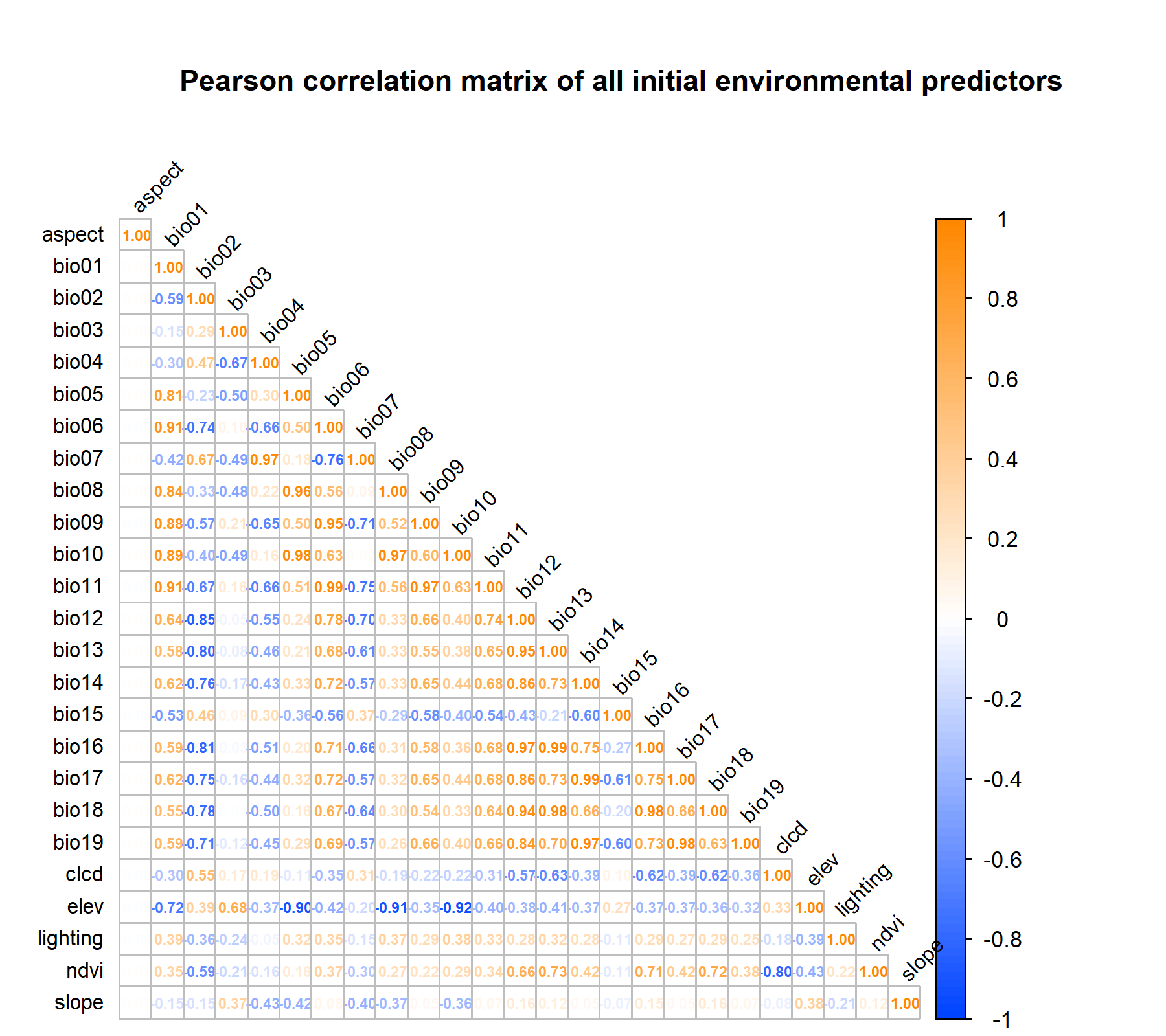

Supplement: Supplementary file 1 [file insects-17-00686-s001.zip › 04Picture/Corr_All_Vars_Clean.png]

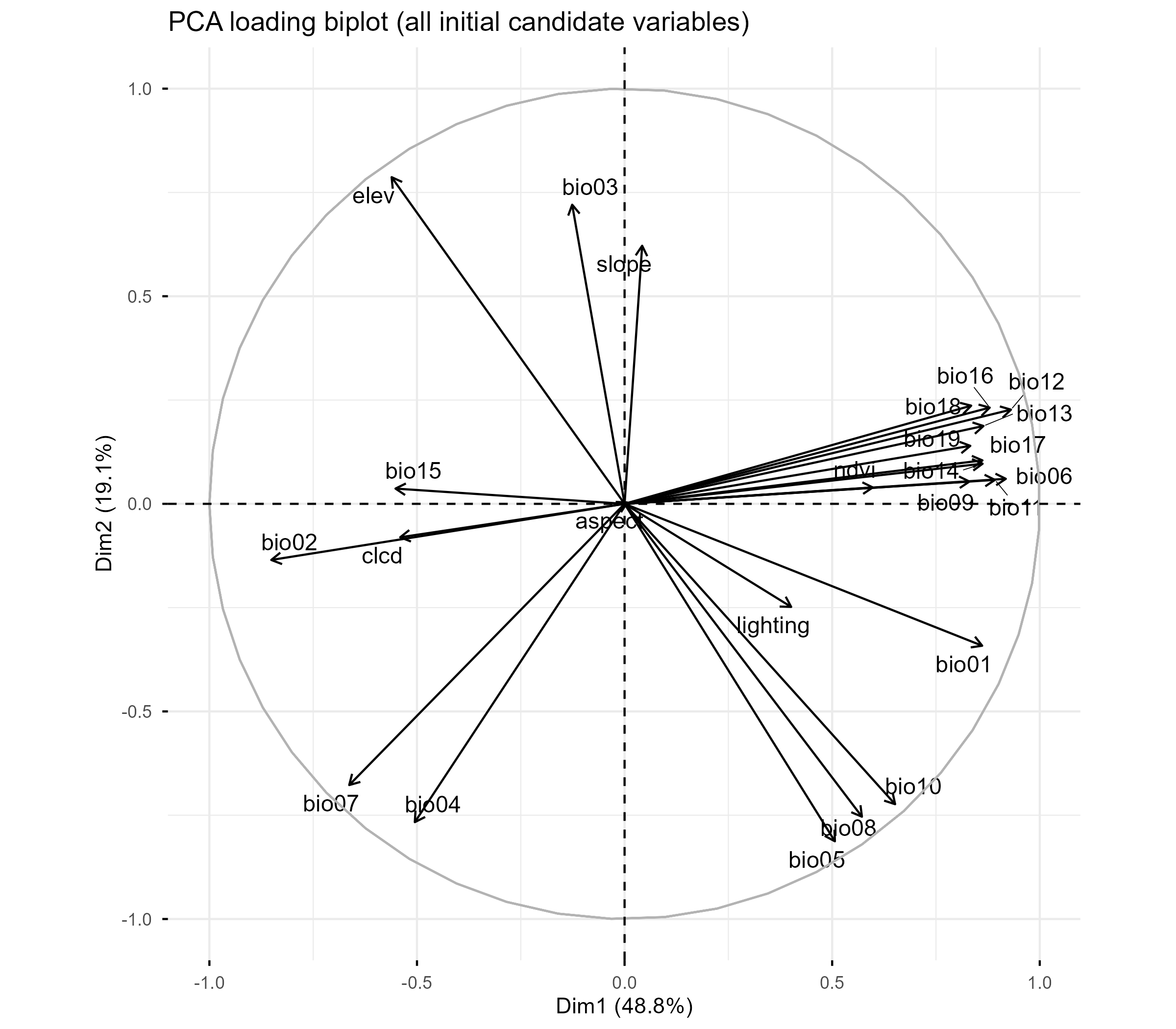

Supplement: Supplementary file 1 [file insects-17-00686-s001.zip › 04Picture/PCA_All_Vars.png]
